# Evaluation of “Indigenous Absorbed ELISA Kit” for the Estimation of Seroprevalence of *Mycobacterium avium* Subspecies *paratuberculosis* Antibodies in Human Beings in North India

**DOI:** 10.5402/2011/636038

**Published:** 2011-05-23

**Authors:** A. V. Singh, S. V. Singh, D. K. Verma, R. Yadav, P. K. Singh, J. S. Sohal

**Affiliations:** ^1^Veterinary Microbiology Laboratory, Animal Health Division, Central Institute for Research on Goats, Makhdoom, P.O. Farah District, Uhar Pradesh, Mathura 281122, India; ^2^Department of Biotechnology, Doon (PG) Paramedical College, Harakland Dehradun 248001, India; ^3^Madhav Institute of Technology and Sciences, Gwalior Madhya Pradesh, India

## Abstract

In present pilot study aimed to estimate, presence of *Mycobacterium avium* subspecies *paratuberculosis* (MAP) antibodies in the human serum samples originating from North India using “Indigenous absorbed ELISA kit” (ELISA kit). The phase I, “ELISA kit” was optimized using protoplasmic antigen from native isolate of MAP “Indian Bison type” recovered from the biopsies of Crohn's disease patients. The phase II, sensitivity and specificity of the kit were estimated as 40.0 and 83.3%, respectively, when evaluated in 40 human serum samples (5 Crohn's disease and 22 ulcerative colitis patients and 13 healthy human subjects) with defined MAP status with respect to stool culture. Seroprevalence of MAP antibodies was higher in CD patients (80.0%) as compared to ulcerative colitis patients (4.5%) and normal human subjects (15.3%). The phase III, seroprevalence of MAP antibodies was estimated as 23.4%, on the basis of the screening of 452 human serum samples (without history) from different geographical regions of North India. Region-wise, 34.0, 33.3, 32.8, 25.0, 23.0, 17.7, and 12.5% samples were positive from the states of Punjab, Uttarakhand, New Delhi, Himachal Pradesh, Haryana, Uttar Pradesh and Jammu and Kashmir, respectively. Study reported moderately higher presence of MAP antibodies in human population, which necessitates programs to reduce the bioburden of MAP in the environment and in animal population.

## 1. Introduction

*Mycobacterium avium* subspecies *paratuberculosis* (MAP), the cause of Johne's disease (JD), has emerged as major pathogen of concern for human health worldwide and has also been associated with Crohn's Disease (CD) in human beings [1–3]. CD is a chronic incurable inflammatory bowel disease (IBD) of gastrointestinal tract (GIT) involving mesenteric and regional lymph nodes and resulting in chronic segmental inflammation that most commonly involves distal ileum or proximal colon, though lesions can occur at any location throughout the GIT [1]. Association of MAP with cases of CD has been supported by frequent isolation of MAP in significantly higher number of CD patients than patients with other bowel disease syndromes and healthy controls [2, 3]. Studies have also shown that like animal paratuberculosis, MAP infection in humans is systematic [3, 4]. PCR, in situ hybridization, and other molecular tools successfully detected MAP DNA in the tissues and blood samples of CD patients [5–7]. Immunological studies using specific, highly purified recombinant antigens also supported the association between MAP infection and cases of CD [8–10]. In the developed countries, “commercial ELISA kits” employed for the detection of MAP antibodies in animals have been successfully adopted for the screening of human serum samples [11, 12]. “Indigenous ELISA kit,” developed in India, was significantly superior when compared with imported “commercial ELISA kits” for the screening of animals [13, 14]. Kumar et al. [15] reported that antigens originating from host-/species-specific MAP had better sensitivity and specificity. MAP is endemic in the domestic livestock population of the country [16–18]. Chances of human exposure to MAP infection are mainly through food chain [19]. Recently, Singh et al. [20] reported high prevalence of MAP in the “animal health care” workers and CD patients. However, in view of the lack of “indigenous diagnostic kits” and reagents, information on the prevalence of MAP in IBD patients (consisting of ulcerative colitis and Crohn's disease) and 1.2 billion human population of the country is limited. The study employed “indigenous absorbed ELISA kit,” based on protoplasmic antigen from native “Indian bison type” MAP genotype recovered from biopsies of CD patient (A 46), for the estimation of sero-prevalence of MAP antibodies in the human population of North India.

## 2. Material and Methods

The study was conducted in three phases. 

### 2.1. Phase I: Optimization of “Indigenous Absorbed ELISA Kit” 

#### 2.1.1. Preparation of Antigen

Semipurified “protoplasmic antigen” (PA) was prepared from “Indian Bison Type” strain (A46) of MAP recovered from the biopsies of CD patient [20] in fourth passage level. MAP was subpassaged in 7–10 slants of HEY medium with mycobactin J at 37°C for 8 months. Growth was harvested, washed and sonicated at 100 W (15 Hz) for 20 min in ice slurry giving 20 cycles of 30 s rest. Sonicate was centrifuged at 9727 g for 30 min at 4°C using Biocentrifuge. Supernatant was dispensed in aliquots of 0.5 and 1 mL and stored at −20°C till further use. A portion of aliquots was used for protein measurement as per Lowry et al. [21].

#### 2.1.2. “Absorbed ELISA Kit”

Presently, country lacks “indigenous kits”, for the screening of either animal or human serum samples. In the present study, “Indigenous absorbed ELISA kit” standardized as per Milner et al. [22] was employed. Optimum concentration of antigen, serum, and second antibody (conjugate) was determined by checkerboard analysis (PA 0.1 *μ*g per well; serum dilution 1 : 50; Rabbit anti-human horseradish peroxidase 1 : 8000). To reduce the background colour and nonspecific binding of protein, optimum blocking agent concentration was standardized using signal-to-noise ratio. Since skimmed milk (3%) in PBS had highest noise ratio, therefore it was employed as blocking agent in the “Indigenous Absorbed ELISA kit”.

#### 2.1.3. Test Protocol

The 0.1 *μ*g of semipurified protoplasmic antigen (PA) in 100 *μ*L of carbonate bicarbonate buffer (pH 9.6) was used for coating duplicate wells of flat-bottom 96 well ELISA plate (Grenier bio-one). Plates were incubated overnight at 4°C followed by washing thrice with washing buffer, PBST (PBS with 0.05% Tween 20). Blocking was done by 200 *μ*L of 3% skimmed milk in PBS and incubated at 37°C for 1 h. After incubation, plates were washed thrice with PBST and then 100 *μ*L of test serum, preabsorbed in PBST containing 1% BSA and 2 mg/mL *Mycobacterium phlei* for overnight at 4°C as per method of Klausen et al. [23], were added to duplicate well and incubated for 2 h at 37°C. After incubation, three washings (5 minutes each) were given with PBST, and 100 *μ*L of optimally PBS-diluted (1 : 8000) conjugate (Sigma) in was added to all the wells. Plate was incubated for 1 h at 37°C and then washed three times with PBST. Finally, 200 *μ*L of freshly prepared substrate (*ortho*-phenylene diamine dihydrochloride-(OPD)), 5 mg per plate in substrate buffer (pH 5.0), was added to each well. Following incubation (in dark) for 20 min at room temperature, absorbance was read at 450 nm in ELISA reader without the addition of stop solution (5 N H_2_SO_4_). Blank, positive, and conjugate controls were also run along with serum samples in each plate. OD values of positive and negative controls were 0.584 and 0.184, respectively. OD values of test samples were transformed to S/P ratio as per Collins [24], and samples in positive ratios, corresponding with “strong positive” category of Collins [24], were considered as positive for MAP infection.

### 2.2. Phase II: Sensitivity and Specificity of “Indigenous Absorbed ELISA Kit”

Sensitivity and specificity of “the kit” were evaluated as per Arizmendi and Grimes [25] using 40 human serum samples (5 CD and 22 UC patients and 13 healthy human subjects) with known MAP culture record. Samples from CD and UC patients were collected from the Department of Gastroenterology, All India Institute of Medical Sciences (AIIMS), New Delhi and Asopa Hospital and Research Center, Agra, UP, India while samples from healthy human subjects were from blood donors at Aastha Pathology Laboratory, Agra, UP, India. Performance of the kit with respect to stool culture was compared by calculating Kappa Scores (Proportional Agreement) as per Altman [26] (0<, poor; 0.0–0.20, slight; 0.21–0.40, fair; 0.41–0.60, moderate; 0.61–0.80, substantial and 0.81–100, almost perfect).

### 2.3. Phase III: Serological Survey (Serum Samples)

Serum samples collected randomly from 452 human beings (329 males and 123 females) in the northern region of the country, between 2004 and 2008, were screened for the MAP antibodies. Geographically, serum samples were obtained from human beings belonging to the states of Uttar Pradesh (180), Rajasthan (47), Jammu and Kashmir (16), Uttra-khand (12), Himanchal Pradesh (2), Punjab (50), Haryana (65), and New Delhi (70) in North India. These serum samples were obtained from human subject without known disease history for MAP antibodies and were therefore considered normal. Serum samples were screened using “indigenous absorbed ELISA kit.” For the reproducibility of the results and to reduce well-to-well and plate-to-plate variations, all tests were performed using four known positive and three negative control serum samples obtained from the earlier studies [20].

## 3. Results

### 3.1. Sensitivity and Specificity of “Indigenous Absorbed ELISA Kit”

To estimate sensitivity and specificity of “Indigenous Absorbed ELISA kit,” culture and ELISA tests were compared on stool and serum samples of 40 human beings. Two tests had 72.5% agreement (positives + negatives). Of the 40 samples screened, 8.3% were positive in the above two tests. The 15.0 (6) and 12.5% (5) samples were positive exclusively in culture and ELISA kit, respectively. There was substantial perfect agreement (proportion agreement = 0.72, kappa value = 0.24) between the two tests. Sensitivity and specificity of indigenous ELISA test were 40.0 and 83.3%, respectively.

### 3.2. Prevalence of MAP Antibodies in Inflammatory Bowel Disease Patients (CD and UC) and Normal Human Subjects

Of the 40 human serum samples (CD and UC patients and normal human beings) used in optimization of “Indigenous absorbed ELISA kit”, 17.5% were positive for MAP antibodies. Prevalence of MAP was higher (18.5%) in inflammatory bowel disease (CD and UC) patients as compared to normal human subjects (15.3). However, individually, 80.0, 4.5, and 15.3% serum samples were positive from CD and UC patients and normal human beings, respectively (Table 1).

### 3.3. Seroprevalence of Anti-MAP Antibodies in Human Beings from North India

Of the 452 human serum samples screened from North India, 23.4% were positive for anti-MAP antibodies (Table 2). Seroprevalence of MAP antibodies was higher in females (31.7%) as compared to male population (20.3%) in North India. State-wise, the highest seroprevalence was found in samples from Punjab (34.0%), followed by Uttarakhand (33.3%), New Delhi (32.8%), Himachal Pradesh (25.0%), Haryana (23.%), Uttar Pradesh (17.7%), and Jammu and Kashmir (12.5%).

## 4. Discussion

In the present study, “Indigenous absorbed ELISA kit” based on native isolate of MAP of “human origin” was standardized to detect MAP antibodies in IBD (CD an UC) patients and to estimate seroprevalence of MAP infection in human beings from North India. ELISA was a rapid and cost-effective alternative to organism detection and can detect low concentration of antibodies in early and late stages of the MAP infection. Optimum antigen concentration was 0.1 *μ*g/well as compared to 1.0 *μ*g/well used by Molina et al. [27]. In the present study, 1 : 50 serum dilution was used as compared to 1 : 25 (lower) serum dilution used by Molina et al. [27] for the detection of MAP antibodies in animals. The use of lower concentration of native antigen and higher dilution of serum in the “indigenous absorbed ELISA kit” may be due to the higher pathogenicity of Indian MAP strain (“Indian bison type”) in animals [28]. Similar conditions were used for the screening of human serum samples. To reduce background noise of proteins, 3% skimmed milk was found to be optimum blocking agent as compared to 5 and 10% skimmed milk and 3, 5, and 10% bovine serum albumin. Johnson et al. [29] also reported the superiority of skimmed milk as blocking agent in comparison to BSA. 

Cutoff value was calculated by sample-to-positive (S/P) ratio [24], instead of average OD of negative samples ± 2 SD [15, 18, 22]. Several researchers advocated quantitative evaluation of OD values or S/P ratios, rather than relying solely on positive or negative classifications defined by a single cutoff value [30, 31]. Studies reported relatively consistent increase in likelihood ratios as the optical density and S/P ratios increased relative to both concurrent fecal culture result and known infection status [32, 33]. Other researchers have also used S/P ratio as cutoff value for the detection of MAP antibodies in human [11, 12] and animals [16, 31] samples. 

The presence of MAP antibodies was higher in CD patients (80.0%) as compared to UC patients (4.5%) and normal human subjects (15.3%) using “Indigenous absorbed ELISA kit,” (Table 1). Higher presence of antibodies in CD and UC patients indicated exposure to MAP. Other researchers [10, 11] have also reported higher presence of MAP in CD patients as compared to UC patients and control subjects. The lower presence of antibodies in UC patients as compared to healthy controls may be attributed to the altered immune response due to the use of immunosuppressive drugs for their treatment [11]. Unlike present findings, 35.0% seropositivity rates for all the groups in a population-based study were reported by Bernstein et al. [12] and there was no difference in the rate of positive samples of CD and UC patients, and healthy controls or nonaffected siblings. 

Sensitivity and specificity of “Indigenous Absorbed ELISA kit” with respect to stool culture were 40.0 and 83.3%, respectively. Slightly lower specificity of the ELISA kit may be attributed to the presence of cell-wall-deficient (CWD) forms of MAP bacilli in human beings. CWD colonies are very difficult to culture on HEY medium. “Indigenous absorbed ELISA kit” had comparable sensitivity and specificity with respect to imported “commercial ELISA kits” when employed to screen serum samples of cattle, goat, and sheep origin (unpublished data). Therefore, “Indigenous Absorbed ELISA kit” may be employed for the screening of anti-MAP antibodies in humans and animals. 

The new “Indigenous ELISA kit” facilitated the screening of large number of human serum samples, and seropresence of MAP antibodies in human beings from North India was 23.4%. A previous study reported 38.0% prevalence of MAP in human subjects from Agra region using “Indigenous Un-absorbed ELISA kit” [20]. Marginally lowered prevalence of MAP antibodies in the present study may be due to the pre-adsorption of test serum with *M. phlei* [34]. Female subjects (31.7%) had higher presence of MAP antibodies as compared to male subjects (20.3%). This may be attributed to the poor sanitation, lowered nutritional status, and additional physical stress (pregnancy, parturition, lactation, etc.) on females, as reported in animals [35]. The presence of antibodies against MAP may represent exposure and not necessarily infection. Therefore, high prevalence of MAP in human beings may be due to the higher bioburden of MAP in the environment [36] and/or the presence of MAP in the food chain [19, 37, 38]. Bernstein et al. [12] also reported higher (33.6%) seroprevalence of MAP in the population of Manitoba by adapting “commercial ELISA kit,” initially developed for the screening of animals for JD. Reconciled findings of present study and Bernstein et al. [12] indicated that Manitobans are simply more exposed to MAP than population of North India or that they have a different genetic ability to mount a response to the infection. Within states in India, seropresence of MAP in human beings ranged from 12.5 to 34.0% (Table 1). Higher sero-presence of MAP antibodies may not be conclusive for MAP infection in the human population of North India. However, the detection of MAP antibodies in higher numbers of human samples indicated higher exposure of human population to MAP infection. Therefore, the control of MAP infection in source population (animals), will be required to reduce the exposure of human population.

## Figures and Tables

**Table 1:** Screening of serum samples collected from IBD patients by indigenous “absorbed ELISA kit.”

Human subjects		Diagnostic center	Samples	Positives

	CD patients	AIIMS, New Delhi	5	4 (80.0%)

IBD patients	UC patients	Asopha Hospital and Research Center, Agra	22	1 (4.5%)
	Total		27	5 (18.5%)

Healthy human subjects*		Astha pathology, Agra	13	2 (15.3%)

Grand total			40	7 (17.5%)

*Healthy blood donors.

**Table 2:** Screening of human serum samples against anti-MAP antibodies by indigenous absorbed ELISA kit.

Region	No. of samples	Positive	% positive
Uttar Pradesh	180	32	17.7
Rajasthan	47	10	21.2
Jammu and Kashmir	16	2	12.5
Uttrakhand	12	4	33.3
Himanchal Pradesh	12	3	25.0
Punjab	50	17	34.0
Haryana	65	15	23.0
New Delhi	70	23	32.8

Total	452	106	23.4
